# Small Nucleolar RNAs and Their Comprehensive Biological Functions in Hepatocellular Carcinoma

**DOI:** 10.3390/cells11172654

**Published:** 2022-08-26

**Authors:** Xiaoyu Liu, Wan Xie, Silu Meng, Xiaoyan Kang, Yuhuan Liu, Lili Guo, Changyu Wang

**Affiliations:** 1 Department of Obstetrics and Gynecology, Tongji Hospital, Tongji Medical College, Huazhong University of Science and Technology, Wuhan 430030, China; 2 Cancer Biology Research Center, Tongji Hospital, Tongji Medical College, Huazhong University of Science and Technology, Wuhan 430030, China

**Keywords:** snoRNAs, HCC, HBV, HCV, NAFLD, NASH

## Abstract

Small nucleolar RNAs (snoRNAs) are a class of highly conserved, stable non-coding RNAs involved in both post-transcriptional modification of RNA and in ribosome biogenesis. Recent research shows that the dysfunction of snoRNAs plays a pivotal role in hepatocellular carcinoma (HCC) and related etiologies, such as hepatitis B virus (HBV), hepatitis C virus (HCV), and non-alcoholic fatty liver disease (NAFLD). Growing evidence suggests that snoRNAs act as oncogenes or tumor suppressors in hepatocellular carcinoma (HCC) through multiple mechanisms. Furthermore, snoRNAs are characterized by their stability in body fluids and their clinical relevance and represent promising tools as diagnostic and prognostic biomarkers. SnoRNAs represent an emerging area of cancer research. In this review, we summarize the classification, biogenesis, activity, and functions of snoRNAs, as well as highlight the mechanism and roles of snoRNAs in HCC and related diseases. Our findings will aid in the understanding of complex processes of tumor occurrence and development, as well as suggest potential diagnostic markers and treatment targets. Furthermore, we discuss several limitations and suggest future research and application directions.

## 1. Introduction

Primary liver cancer is the sixth most common malignancy and the third leading cause of cancer-related mortality worldwide, with approximately 906,000 new cases and 830,000 deaths in 2020 [1]. Hepatocellular carcinoma (HCC) is the most common histological subtype of liver cancer, accounting for 90% of all cases [2]. HCC is commonly caused by chronic infection with hepatitis B virus (HBV), hepatitis C virus (HCV), high alcohol consumption, or non-alcoholic fatty liver disease (NAFLD) [3]. Currently, in addition to the most common and effective treatments for HCC, such as liver resection, liver transplantation, transarterial chemoembolization, and local ablation with radiofrequency, some novel therapeutic approaches, such as the use of monoclonal antibodies, immune-checkpoint inhibitors, and tyrosine kinase inhibitors, provide substantial survival benefits for patients [2]. Although substantial advances have been made in all areas and preliminary results are encouraging, overall treatment outcomes remain unsatisfactory.

SnoRNAs are a specific class of small, abundant, and stable non-coding endogenous RNAs with a length of 60 to 200 nucleotides that localize in the nucleolus [4]. The classical function of snoRNAs is to guide the chemical modification of ribosomal RNAs (rRNAs), small nuclear RNAs (snRNAs), and transfer RNAs (tRNAs) [5,6]. The advancement of high-throughput RNA sequencing technology in recent decades has led to the identification of many differentially expressed snoRNAs in various disorders. SnoRNAs have gained increasing recognition and have been proven to play a critical role in maintaining normal physiological function and the pathogenesis and progression of diseases, including cancers [7,8,9]. They can operate individually or together to impact carcinogenesis [10]. Furthermore, it is becoming obvious that snoRNAs exert broader biological functions in non-canonical ways than previously thought [11]. Research investigating snoRNAs has revealed a new avenue of tumorigenesis and has shed new light on the diagnosis and treatment of HCC. In this review, we provide a brief overview of the structure and function of snoRNAs and focus on the recent progress of snoRNAs in HCC and associated diseases.

## 2. Biogenesis and Structure of SnoRNAs

**Figure 1:**
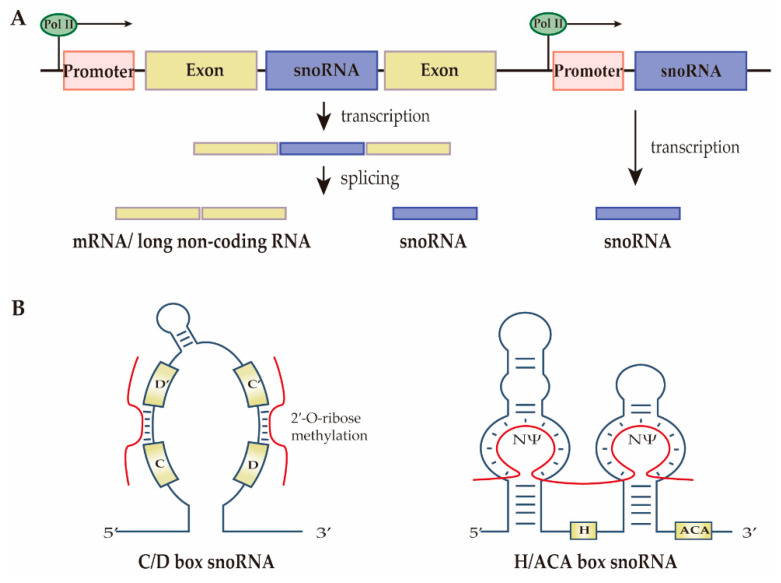
Biogenesis and structure of small nucleolar RNAs (snoRNAs). (**A**) SnoRNA biosynthesis. Most identified snoRNA genes are located in intronic regions of protein-coding genes or long non-coding sequences. They are transcribed by RNA polymerase II (Pol II) and are released from their transcripts after splicing. A small subset of snoRNAs is produced from single genes with independent promoters. (**B**) SnoRNA structure. C/D box snoRNAs have two conserved sequences, namely box C (RUGAUGA) and box D (CUGA). The upstream of box D’/D is complementary to the target RNAs and guides the 2′-O-ribose methylation. H/ACA box snoRNAs contain conserved H box (ANANNA) and ACA box. They also have two pseudouridylation (NΨ) pockets complementary to the target RNAs to direct their pseudouridine modifications.

According to the snoDB database, 2064 snoRNAs have been identified as of 2020 [4]. Almost all snoRNAs are produced from introns of protein-coding genes or non-coding genes after special selective splicing. However, a small portion of snoRNAs also derive from independent transcripts (Figure 1A) [12]. SnoRNAs have highly conserved sequences and specific secondary structures. All snoRNAs can be divided into two main families, box C/D snoRNA (SNORD) and box H/ACA snoRNA (SNORA), based on their common sequences, structural characteristics, and the small nucleolar ribonucleoproteins (snoRNP) they form (Figure 1B) [13].

The SNORD family contains a conserved sequence box C (RUGAUGA) and box D (CUGA), forming a Kink-turn motif responsible for guiding 2′-O-ribose methylation of their RNA targets. Additionally, most SNORDs have less conserved C’ and D’ box motifs. The C/D box snoRNAs perform their function by direct formation of snoRNP complexes with core proteins, namely 15.5K, NOP56, NOP58, and fibrillarin proteins. The SNORA family contains an H-box (ANANNA) and a trinucleotide ACA box, which exhibits a ‘hairpin-hinge-hairpin-tail’ secondary structure that guides the pseudouridine (Ψ) modifications. The H/ACA snoRNPs comprise four core proteins, namely the pseudouridine synthase dyskerin, Nhp2, Nop10, and Gar1, respectively [14]. Small Cajal body associated RNAs are a particular subset of snoRNAs named for their subcellular localization. They have characteristic box C/D and box H/ACA sequence motif features, structures, and corresponding functions [15].

## 3. Biological Functions of SnoRNAs

The typical functions of snoRNAs are the 2’-O-ribose methylation and pseudouridylation of rRNAs, tRNAs, and snRNAs. SnoRNAs carrying specific sequences that align in the antisense orientation to rRNAs or snRNAs are called guide snoRNAs, while other snoRNAs lacking apparent complementarity to targeted RNAs are called orphan snoRNAs, accounting for 17% of all snoRNAs [14]. Orphan snoRNAs cannot guide nucleotide modification but can target their unique potential RNAs and proteins, suggesting regulatory functions in noncanonical pathways [16].

SnoRNAs also participate in complex cell biological processes. In addition to RNA modification, previous studies have identified various functions of snoRNAs, such as pre-rRNA and messenger RNA (mRNA) processing, telomere synthesis, and the maintenance and opening of chromatin [17,18]. SnoRNAs can also serve as precursors for microRNAs, piwi-interacting RNAs, and snoRNA-derived RNAs (sdRNAs) to display new functions after a series of splicing [18,19,20,21].

Until recently, the role of snoRNAs in cancer progression has also gradually been revealed. SnoRNAs can act as oncogenic or tumor suppressor regulators through various mechanisms. They can cause ribosomal RNA modifications to disrupt the protein translation process in myeloid leukemogenesis [22]. A proportion of snoRNAs bind and activate poly (ADP-ribose) polymerases-1 and further induce ribosomal DNA transcription, ribosome biogenesis, and DNA damage repair to promote tumorigenesis [23]. Furthermore, numerous studies have identified snoRNAs as upstream and downstream components in various tumor signaling pathways that govern cell fate. In breast cancer, up-regulated snoRNAs can serve as fibrillarin binding oncogenes to block p53 activation, while SNORD50A and SNORD50B act as tumor suppressor genes, whose deletions lead to p53 degradation to promote tumor progression [24,25]. Overexpression of SNORA71A stimulates epithelial-mesenchymal transition (EMT) by regulating the MAPK/ERK pathway in lung cancer [26]. Ectopic expression of SNORA72 activates stem cell transformation of ovarian cancer cells through the Notch1/c-Myc pathway [27]. SnoRNAs can also act as the downstream molecule of p53 and MYC responsible for tumorigenesis [28,29]. Furthermore, a large group of sdRNAs is significantly associated with the characteristics of the tumor-immune microenvironment, and some of the sdRNAs function similar to the microRNAs involved in cancer progression and chemoresistance [30,31].

## 4. SnoRNA Expression Profiling in HCC and Associated Diseases

**Figure 2:**
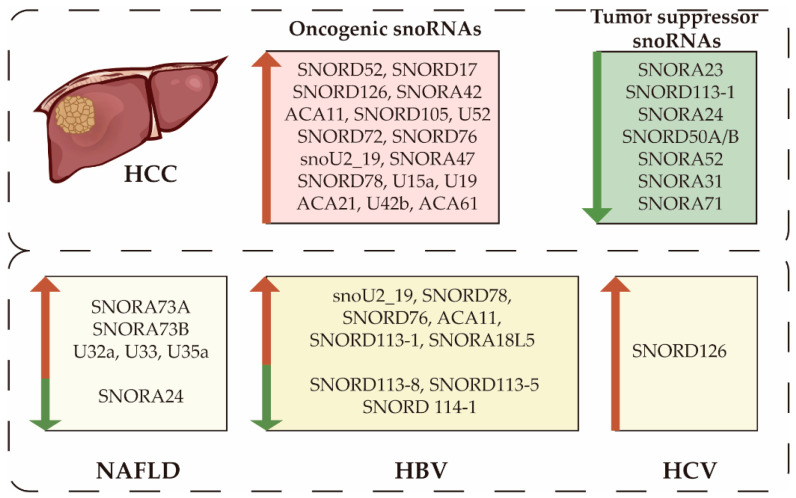
Dysregulated snoRNAs in hepatocellular carcinoma (HCC) and associated diseases.

In many studies based on microarray and whole genome transcriptome sequencing platforms, snoRNA expression levels are dysregulated in HCC (Figure 2). Yang et al. observed an overall up-regulation of snoRNAs according to The Cancer Genome Atlas database of 372 HCC and 50 non-tumor tissues. They identified 54 up-regulated and 14 down-regulated snoRNAs enriched in the ribosome pathway, the cell cycle, and DNA replication [32]. Liang et al. analyzed two HCC cohorts from the Gene Expression Omnibus database, in which 54 differentially expressed snoRNAs were identified [28]. Using high-throughput small RNA sequencing of six pairs of HCC tumor tissues and corresponding noncancerous liver tissues, Wang et al. identified 10 up-regulated and 7 down-regulated snoRNAs [33]. Recently, partial snoRNA has been identified as a tumor suppressor gene or an oncogene by functional studies. Many studies have been conducted investigating the pathological mechanisms of single dysregulated snoRNAs, which we will describe in the following sections.

From an etiologic perspective, multiple diseases are responsible for liver cancer, including long-term infection with HBV or HCV, abnormal lipid metabolism, alcoholic liver disease, and NAFLD [34]. SnoRNAs have been involved in some of these HCC-associated diseases (Figure 2). Duplication of SNORA18L5 increases the risk of HBV-related HCC, and SNORD126 promotes HCV infection [35,36]. SnoRNA U32a, U33, and U35a act as critical mediators in metabolic stress, and their loss induces lipotoxicity resistance [37].

From a histopathological perspective, hepatocarcinogenesis is a long and multi-step process that starts the typical pathological process of chronic liver injury and inflammation and then progresses to cirrhosis and even liver cancer [38]. Koduru et al. obtained publicly available small RNA sequencing data from the National Institutes of Health’s short read archive containing 9 healthy livers, 9 low-grade dysplastic nodules, 6 high-grade dysplastic nodules, 14 cirrhosis, 6 early HCC, and 20 advanced HCC tissues. Differential gene expression analysis showed that three snoRNAs (SNORD115-31, SNORD121B, and SNORA37) were negatively regulated in four types of pathological liver conditions [39]. These findings implicated the involvement of snoRNAs in the overall process of liver injury. However, no significant differences were identified between different pathological processes, which could be due to the small sample size. Regrettably, no further studies have been conducted at each stage.

## 5. Clinical Significance of Altered SnoRNAs in HCC

Given the high rates of recurrence and mortality of HCC after surgical intervention, novel biomarkers for early diagnosis, prognostic evaluation, and tumor classification are urgently needed. Altered snoRNAs were considered potential biomarkers of HCC and prognostic factors to predict recurrence and survival time. Current methods for detecting snoRNA include quantitative real-time PCR, microarrays, RNA-RNA sequencing, and other diagnostic methods based on PCR or sequencing [40,41]. Yang et al. identified 9 snoRNAs as independent prognostic factors (SNORA24, SNORA7, SNORA63, U3_chr8-2, U3_chr9, SNORD19B, hTR, SNORD36C, and U44) and subsequently constructed a prognostic risk score model [32]. The patients were then divided into low and high-risk categories according to their risk scores. Validation assays suggested that the risk of HCC death was much higher for patients in the high-risk group than for those in the low-risk group. Zhuang et al. constructed a prognostic model for the risk of relapse that contained 7 snoRNAs by sequencing HCC tissues and adjacent normal tissues of 283 HCC patients [42]. Several studies of a single snoRNA in HCC patients revealed the clinical significance of snoRNAs. SNORA52, SNORA31, and SNORA71 were down-regulated in HCC and had a significant clinical association with tumor size, lesion number, capsular invasion, degree of tumor, and TNM stage. Lower expression of these three snoRNAs manifested shorter disease-free survival and shorter overall survival (OS) [43,44,45]. SNORD76 and ACA11 were up-regulated in HCC and their high levels were correlated with histological grade, Barcelona Clinic Liver Cancer stage, HBV infection, and portal vein tumor thrombus [46,47]. These differentially expressed snoRNAs may have potential as a prognostic indicator.

A large number of studies have demonstrated the stable presence of snoRNAs in serum, plasma, urine, cell-free saliva, and tissue samples. Several studies confirmed the feasibility of snoRNAs as non-invasive liquid diagnostic and prognostic biomarkers in various cancers [48,49,50]. One study found 38 highly enriched snoRNAs in extracellular vesicles of 4 liver-cancer cell lines, and 9 of them displayed high levels of expression [51]. In plasma from patients with HCC, snoRNAs accounted for a large proportion of differentially expressed genes [52]. Although there was no evidence that these snoRNAs were unique to HCC given their high abundance and heterogeneity, it hinted at their potential as diagnostic biomarkers. ASO and LNA are common approaches for targeting snoRNAs. The European Medicines Agency and the US Food and Drug Administration have both approved the ASO drug Nusinersen for the treatment of spinal muscular atrophy [53]. However, to date, there have been no clinical trials in HCC.

## 6. SnoRNAs and Liver Carcinogenesis

**Figure 3:**
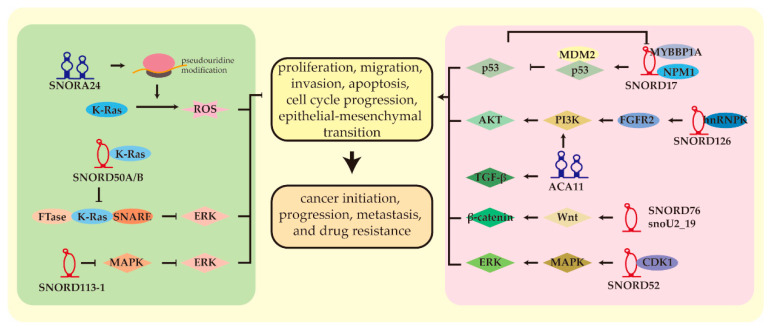
Mechanisms of snoRNAs in HCC. Abnormal expression of snoRNAs could lead to various pathophysiological changes. They could regulate a variety of signaling pathways by binding or releasing target proteins. Aberrant expression of snoRNAs in HCC could activate PI3K/AKT, Wnt/β-catenin, TGF-β, MAPK/ERK pathways while inhibiting the p53 pathway. They played a critical role in cellular processes, including proliferation, migration, invasion, apoptosis, cell cycle progression, and epithelial-mesenchymal transition, which were critical for cancer initiation, progression, metastasis, and drug resistance.

Several studies have shown that snoRNAs act as a tumor promoter or suppressor in HCC (Table 1). SnoRNAs could significantly affect pathophysiological processes, including tumor initiation, progression, metastasis, and drug resistance [28,54,55]. Some molecular mechanisms have been intensively investigated (Figure 3). Here, we reviewed the relevant literature on HCC-related snoRNAs.

### 6.1. From Ribosome Biogenesis to Carcinogenesis

SnoRNAs are responsible for nucleotide modification of rRNAs; disorders of ribosomal activity have been shown to transform normal healthy cells into neoplastic cells [65]. In HCC, is it possible that dysregulations of snoRNAs influence cancer initiation and progression through effects on ribosomes? McMahon et al. provided a response to this hypothesis [54].

Oncogene-induced cellular senescence (OIS) is a vital cellular defense response to the arrest of malignant neoplasms. It usually occurs in cells expressing activated oncogenes to prevent malignant progression [66]. By overexpressing activated oncoprotein RASG12V to stimulate primary human skin fibroblasts, McMahon et al. found that overall snoRNA expression increased, while the levels of protein synthesis were instead reduced [54]. This was the process in which cells underwent OIS to overcome oncogene-induced malignant transformations. Among these snoRNAs, SNORA24 was up-regulated in RAS-induced senescence but significantly down-regulated in HCC tissues. In a mouse liver model, early senescence was activated by RAS but was not accompanied by tumorigenesis. When using locked nucleic acid (LNA) to degrade SNORA24, senescence progress was inhibited, and malignant transformation was initiated by synergy. A low level of SNORA24 played a critical role in tumor initiation and progression and in the ability to evade the tumor-suppressive defense mechanism conferred by OIS. SNORA24 was mainly responsible for pseudouridine modifications of two sites in the 18S rRNA of the small 40S subunit: uridine 609 and uridine 863 [67,68]. SNORA24 influenced multiple aspects of mRNA translation. Translation is a complex and dynamic multistep process, including initiation, elongation of the polypeptide chain, and termination [69]. Decoding, peptidyl transfer, and translocation coordinate with each other to ensure translation accuracy. Several of these processes were altered in SNORA24 knockdown HCC cells [54]. The dynamics of ribosomes lacking SNORA24-guided modifications changed, which showed a powerful ability to select tRNA and showed a preference for different states of tRNAs during decoding compared to control cells. SNORA24 appeared to be dispensable for global protein levels, but its deficiency impaired translation fidelity in a codon-specific manner. The Ψ609 modified by SNORA24 was located in the decoding center of the ribosome, a site that is directly involved in the recognition of codon anticodon during the decoding process of the ribosome [70]. Ribosome defects disrupted the precision of mRNA decoding, leading to errors in the decoding of many codons, including the stop codon [54]. Finally, under the combined effect of efficient selection of aa-tRNA, conformational dynamic differences, and decreased decoding precision, the loss of SNORA24 resulted in specific OIS-related mRNA translation errors in HCC, allowing liver cells to escape RAS-induced senescence and continue malignant transformation, thus contributing to HCC.

### 6.2. Noncanonical Functions of SnoRNAs in HCC

Although snoRNAs lacking complementarity with canonical rRNA targets are unable to regulate post-transcriptional modifications, evidence has revealed that they function by binding to unconventional targets. Several studies have comprehensively explored the molecular mechanism involved in the progression of HCC involving snoRNAs and nonclassical snoRNPs.

SNORD17 has been identified as an oncogene in a variety of human tumors, including HCC, whose level in plasma exosomes has emerged as a diagnostic and prognostic marker for cervical cancer [28,71,72]. SNORD17-derived SdRNAs were associated with the level of CD8+ tumor infiltrating lymphocytes [30]. In HCC, SNORD17 conferred a higher capacity for proliferation, resistance to apoptosis, and cell cycle progression in vitro, as well as a metastatic capacity of lungs in vivo against cancers [28]. The p53 proteins are essential for this process. SNORD17 could specifically bind to nucleophosmin1 (NPM1) and Myb-binding protein 1A (MYBBP1A) to regulate the p53 pathway. NPM1 accumulates primarily in the nucleolus and was involved in ribosome biogenesis [73]. Different cellular stresses such as DNA damage and proteasome inhibition could make NPM1 translocate from nucleoli to nucleoplasm, where it was bound to human murine double minute 2 (MDM2), an oncogene that acted as a ubiquitin ligase for proteasomal degradation of p53. Relocalization of NPM1 competed for MDM2 to block its domain with E3 ligase activity, as well as caused dissociation of MDM2-p53 complexes and eventually inhibited the degradation of p53. MYBBP1A was also a nucleolar protein that could be redistributed from nucleoli to nucleoplasm under nucleolar stress, where it interacted with p53 and enhanced p300-mediated p53 acetylation by promoting p53 tetramerization, allowing p53 to be more activated [74]. In HCC, NPM1 and MYBBP1A translocated to the nucleoli and colocalized with SNORD17 [28]. Through truncations generated from NPM1 and MYBBP1A that contained nucleotide binding domains, overexpressed SNORD17 was combined with them. The formation of the complex made NPM1 and MYBBP1A anchored to the nucleoli to prevent the combination of NPM1-MDM2-p53 and MYBBP1A-p300-p53. Furthermore, in the promotor region of SNORD17, there was a binding site for p53. An increase in p53 could repress SNORD17 expression mediated by p300. In sum, the high level of SNORD17 in HCC anchored NPM1 and MYBB1A to the nucleoli, thus decreasing the stability and transcriptional activity of p53. This decrease in p53 was able to enhance the expression of SNORD17 in turn. This reciprocal regulation between SNORD17 and p53 ultimately constituted a positive feedback loop that contributed to tumorigenesis and development.

SNORD52 was dramatically up-regulated in HCC and was inversely correlated with a poor prognosis [42]. In vitro and in vivo, it exhibited pro-oncogenic effects on biological behavior and function [56]. The HCC suppressor gene Up-frameshift 1 is an upstream signaling molecule of SNORD52, whose low expression level results in a low level of nonsense-mediated decay [75]. Following SNORD52 transcription, the premature termination codon and exon junction complex are generated, but they cannot be recognized or degraded over time, eventually leading to up-regulation of SNORD52. SNORD52 overexpression interacted with cyclin-dependent kinase 1 (CDK1), an oncogene that drives the S/G2 and G2/M cell cycle transitions in HCC [76]. The study showed that SNORD52 caused less ubiquitination and proteasomal degradation, more phosphorylation, and more stability of CDK1, raising its protein level.

A pancancer study reported that the somatic loss of SNORD50A and SNORD50B is a common event in many types of malignancies [62]. At least 20% of individuals with HCC exhibit such a deletion, which is associated with poor clinical outcomes. K-Ras requires activation and plasma membrane enrichment. Binding farnesyltransferase (FTase) to obtain prenylation is a limiting process [70]. Furthermore, binding to soluble N-ethylmaleimide-sensitive factor attachment protein receptor (SNARE) proteins is required for K-Ras translocation towards the plasma membrane [77,78]. As endogenous inhibitors, SNORD50A and SNORD50B tightly bind KRAS to weaken KRAS binding to the FTase and SNARE proteins, inhibiting the cancer-causing activities [62,78]. In a variety of human cancer cells, frequent deletion of SNORD50A/B releases common K-Ras binding sites, leading to activation of KRAS and its downstream MAPK/ERK pathway.

SNORD126 is significantly overexpressed in HCC and has been correlated with a poor prognosis [57]. Its overexpression significantly increased tumor cell proliferation and resistance to cisplatin, etoposide, and vinblastine [55]. In HCC, SNORD126 combines with a heterogeneous nuclear ribonucleoprotein K that relies on the C’ and D boxes. The complex is then recruited to the promoter of the fibroblast growth factor receptor 2 to promote its transcription to activate the PI3K/AKT pathway.

### 6.3. Other SnoRNAs

Several studies have also described the role of snoRNAs at the cellular level and provided simple insights into the mechanism underlying the oncogenic or tumor suppressor functions. Alterations in tumor biologic behaviors and downstream signaling pathways resulted from dysregulation of snoRNAs in HCC that constituted snoRNA target gene pathways.

SNORD113-1 is not expressed in HCC due to the high methylation of the CpG islands in its upstream region [63]. Low levels are correlated with a poor clinical outcome. However, its deletion did not exert a noticeable effect on global mRNA expression at the transcriptional level, but suppressed HCC tumorigenesis through the MAPK/ERK and TGF-βpathways. SNORA42 is highly upregulated in HCC and correlates with a poor prognosis [58]. It is critical in the development of HCC, and promotes proliferation, migration, invasion, and inhibition of apoptosis. Mechanistically, SNORA42 accelerates the cell cycle progression by interfering with the p53 pathway. HCC samples present high expression of ACA11, which promotes cell growth, migration, and invasion through the PI3K/AKT pathway [46]. Meanwhile, its downstream factor Cyclin D1 is activated to promote cell cycle progression and EMT. Patients with elevated levels of ACA11 are prone to recurrence and shorter OS. Receiver operating characteristic curves analysis demonstrated that ACA11 may represent a potential diagnostic biomarker of HCC with an area under the curve of 0.81. SNORA23 is down-regulated and functions as a tumor suppressor in vitro and in vivo [64]. Activation of the PI3K/AKT pathway is one of the most prevalent oncogenic events in various cancers, resulting in downregulation of SNORA23 in HCC. SNORA23 deficiency inhibits the combination with 28S rRNA and further reduces its 2′-O-methylation. Moreover, SNORA23 reduces the phosphorylation of 4E binding protein 1, a known downstream regulator of the PI3K/AKT/mTOR pathway, to promote tumorigenesis. Single nucleotide polymorphisms in SNORD105 alter susceptibility to HCC [59]. Patients with the GG genotype have a lower risk of developing HCC than those with the AA genotype, due to the lower expression of SNORD105. HCC cells with SNORD105 overexpression gain greater viability and migration ability. LncRNA-LALR1 has been identified as a tumor-promoter upregulated in HCC, which has been shown to bind to SNORD72 and improve its expression [60]. Elevated SNORD72 levels improve the stability of mRNA of DNA-binding inhibitors 2, thus contributing to HCC. SNORD76 and the small nucleolar RNA U2_19 (snoU2_19) operate as oncogenic factors in HCC, promoting proliferation, cell cycle, and invasion by activating the Wnt/β-catenin pathway [33,47]. SNORD76 and SNORA47 promote EMT and facilitate the progression of HCC [61].

## 7. Hepatitis Virus-related Hepatocarcinogenesis

HBV and HCV infections continue to be the leading causes of HCC. HBV infections accounted for 33% of HCC deaths, while HCV infections accounted for 21% from 1990 to 2015 [79]. HBV and HCV can cause chronic hepatitis, cirrhosis, and eventually HCC. HBV contributes to HCC in several ways. In HCCs developed in HBV carriers, HBV genes that integrate with host genomic DNA are typical. Chimeric HBV-human transcripts and genome instability caused by HBV integration induce cancer-related gene expression and activation, driving tumorigenesis [80]. The HBVx protein is also tumorigenic and is involved in the development of HCC [81]. Unlike HBV, HCV does not have the potential to be directly involved in HCC; most HCV-associated HCC events occur in the context of cirrhosis [82].

Several studies have analyzed the clinical relevance between snoRNAs and hepatitis virus infection in patients with HCC. Compared with patients without HBV, HBV carriers have higher expression levels of snoU2_19, SNORD78, SNORD76, ACA11, and SNORD113-1, and lower levels of SNORD113-8, SNORD113-5, and SNORD 114-1 [33,46,47,63,83]. Cao et al. conducted a genome-wide association study on 1583 HBV patients with HCC and 1540 HBV patients without HCC to investigate germline copy number variations during chronic HBV progression to HCC [35]. They found low-frequency duplication on chromosome 15q13.3, a heritable genetic variation common to multiple types of cancers. This site did not encode any proteins but contained the snoRNA SNORA18L5, whose expression was proportional to the gene copy number. Meanwhile, the upregulated expression of H3K4me3 in its promoter region also increased SNORA18L5. Mechanistically, it accumulated 28S and 18S rRNAs maturation to hyperactive ribosome biogenesis. Similar to SNORD17, SNORA18L5 promoted HCC in a p53-dependent manner. SNORA18L5 overexpression in HCC bound to two ribosome proteins, RPL5 and RPL11, in the nucleolus, causing their absence in the nucleoplasm. The reduced combination of MDM2 and RPL5/RPL11 eventually released more MDM2 and increased the ubiquitination and degradation of MDM2-mediated p53. Another study revealed the role of snoRNAs in HCV susceptibility [36]. Several changes in snoRNA expression were observed through high-throughput small RNA sequencing during HCV infection, which contained 40 up-regulated snoRNAs and 13 down-regulated snoRNAs, with SNORD126 being the most significantly down-regulated as the infection progressed both in vitro and in vivo. By evaluating HCV RNA and viral core protein expression levels, the experiments revealed that SNORD126 facilitated HCV entry into the host gene dose-dependently without affecting viral replication or release. SNORD126 promoted the expression and distribution on the cell surface of an HCV entry factor, claudin-1, by activating the PI3K/AKT pathway. The susceptibility to HCV caused by SNORD126 overexpression was reversed when any step in this pathway was disrupted. As stated previously, SNORD126 is up-regulated in HCC while down-regulated here, suggesting that its expression could be dynamic and alters during pathogenesis [55]. During early infection, SNORD126 decreased to resist external infection, and once this process is complete, it may increase to promote cancer initiation.

## 8. Deregulation of SnoRNAs in NAFLD and Nonalcoholic Steatohepatitis

Currently, HBV and HCV infection are still the leading causes of HCC. However, their importance will decrease with the prevalence of HBV vaccination and the development of antiviral therapy. In contrast, metabolic diseases such as metabolic syndrome, type II diabetes, obesity, and nonalcoholic steatohepatitis (NASH)-related liver disease will become more significant in HCC in the coming years [84]. Currently, the epidemiology of HCC has changed from viral hepatitis to NASH, and NAFLD has become the fastest growing cause of HCC worldwide [85]. NAFLD is one of the most common metabolic diseases that covers a variety of pathophysiological processes from nonalcoholic fatty liver to steatohepatitis and can progress to NASH cirrhosis and NASH-related HCC. NAFLD initially exhibits hepatic steatosis and then progresses to serious inflammation and hepatocyte damage [86]. The global incidence of NAFLD is increasing, accounting for 25% of total adults, of which approximately 1.5% to 6.45% have NASH [87]. The annual incidence of HCC in patients with NASH is 5.59 per 1000 person-years, and half of patients can develop HCC without going through cirrhosis [88]. According to a systematic review and meta-analysis of the prevalence of HCC related to NAFLD from its inception to 2022, 15.1% of HCC cases were secondary to NAFLD, and the proportion is increasing [89]. Although the reported incidence is lower than HBV or HCV, the high burden of NAFLD and NASH will contribute to an increase in patients with NAFLD and NASH-associated HCC.

SNHG3 is an overexpressed lncRNA with pro-oncogenic effects in multiple cancer entities, including HCC [90]. Its introns encoded two snoRNAs, SNORA73A and SNORA73B, which have been reported to play a vital role in maintaining cellular cholesterol homeostasis, especially in cholesterol esterification and trafficking [91]. 2E4 is a mutant cell line highly resistant to lipid-induced oxidative stress and cell death, in which the loci encoding SNORA73 and SNHG3 are destroyed. Through a series of SNHG3 and SNORA73 knockdown experiments, one study definitively clarified that SNORA73 exerted a role in response to lipotoxicity, an early event in NASH [92]. Compared to wild type cells, 2E4 cells produced fewer reactive oxygen species and more critical antioxidant glutathione and NADPH when exposed to lipotoxic free fatty acid palmitate. Subsequent experiments proved that the loss of SNORA73 induced increased mitochondrial metabolism, oxidative phosphorylation, and glutathione biosynthesis, but not aerobic glycolysis or the pentose phosphate pathway. Furthermore, the loss of SNORA73 also improved the ability to handle the increased capacity of fatty acid substrates. The same pattern of metabolic alterations and improved lipotoxicity was also found in a liver steatosis mouse model induced by a high-fat diet. SNORA73 was essential for the processing and modification of rRNAs, but the data did not reveal a change in the abundance of rRNAs [93]. mTOR played a central role as a bridge in this process. Studies reported that the mTOR pathway could be activated by impaired rRNA production and it could regulate metabolism [94,95]. Defective processing of rRNA caused by loss of SNORA73 acts as an initiator to activate the mTOR pathway, leading to metabolic reprogramming to counteract lipotoxicity. Another study found that snoRNA U32a, U33, and U35a worked similarly [37]. These three snoRNAs encoded at the L13a ribosomal protein locus could be activated in lipopolysaccharide-induced liver damage and were also involved in oxidative stress. Elimination of these three snoRNAs protected the cell from reactive oxygen species, endoplasmic reticulum stress, and oxidative stress caused by lipotoxicity stimulation in vitro and in vivo. SNORA24, which we previously discussed, was also strongly associated with lipid deposition [54]. Acting as a tumor suppressor, SNORA24 is down-regulated in HCC and its low levels were associated with decreased survival. At the same time, SNORA24 expression levels were inversely correlated with lipid content. In the human HCC cell line HuH7, the suppression of SNORA24 knockdown enhanced lipid droplet formation. Similarly, tumor tissues were pathologically characterized by dramatic accumulation of lipids in the mouse HCC model induced by the combination of RAS and LNA-24. All of these results indicated that the down-regulation of SNORA24 in HCC triggered aberrant lipid metabolism and promoted tumor formation and maintenance. However, it remains to be investigated whether defects in rRNA modification are correlated with dysmetabolic fate.

## 9. Conclusions and Future Perspectives

There are many abnormally expressed snoRNAs in HCC and related diseases such as HBV-associated liver cancer, HCV-associated liver cancer, and NAFLD. Such snoRNAs are involved in HCC initiation, maintenance, metastasis, and drug resistance [28,54,55]. They are also associated with clinicopathological factors and prognosis in HCC. Meanwhile, based on their stability in body fluids, aberrantly expressed snoRNAs have implications for diagnosis and prognosis and hint at the potential for non-invasive screening [52]. The precise mechanisms of snoRNA dysregulation in HCC contain gene deletion, single nucleotide polymorphisms, copy number variations, transcription factors, and DNA methylation [28,35,59,62,63]. These abnormally expressed snoRNAs can serve as oncogenes and tumor suppressor genes. SnoRNAs can cause disturbances in ribosome biogenesis and interfere with the precision of protein synthesis [54]. They can also exert non-classical functions by forming non-canonical snoRNP particles, which can alter target activity, intracellular localization, or interaction capability, thus playing a significant role in tumor development at multiple stages [62]. Abnormal expression of snoRNAs can regulate numerous signaling pathways, including the p53, Wnt/β-catenin, PI3K/AKT, and MAPK/ERK pathways [28,47,63,64]. Several studies have been conducted to investigate the underlying molecular mechanisms of snoRNAs, forming complete signaling transduction mechanisms, as well as synergistic and dynamic regulatory networks in hepatocarcinogenesis.

Despite this progress, many questions remain to be explored. For example, HCC-specific snoRNAs have not been identified. SnoRNA expression changes in different pathological processes and etiologies are still uncertain. No clinical trial of reliable and valid diagnosis and therapy based on snoRNAs has been launched. Future studies need to address these gaps. We expect a deeper understanding of snoRNAs in tumor biology and molecular pathways to be provided in the future. We hope that snoRNAs can open up new frontiers in clinical translation to guide diagnosis and optimization of personalized therapy.

## Figures and Tables

**Table 1:** HCC-related snoRNAs.

snoRNA	Chromosomal Location	Host Gene	Role in HCC	Expression	Sample Size, HCC/Control	Targets	Reference
SNORD52	6p21.33	SNHG32	Oncogene	Up	80/80	CDK1	[56]
SNORD17	20p11.23	SNX5	Oncogene	Up	175/175	NPM1, MYBBP1A	[28]
SNORD126	14q11.2	CCNB1IP-1	Oncogene	Up	30/30	hnRNPK	[55,57]
SNORA42	1q22	KHDC4	Oncogene	Up	60/60	P53, p21	[58]
ACA11	4p16.3	NSD2	Oncogene	Up	92/92	-	[46]
SNORD105	19p13.2	PPAN-P2Rγ11	Oncogene	Up	712/801	PPAN	[59]
SNORD72	5p13.1	-	Oncogene	Up	46/46	ID2	[60]
SNORD76	1q25.1	GAS5	Oncogene	Up	66/66	Fibronectin, vimentin	[47]
snoU2_19	4, 7	-	Oncogene	Up	80/80	β-catenin	[33]
SNORA47	5q13.3	ZBED3	Oncogene	Up	60/60	-	[61]
SNORA24	4q26	SNHG8	Tumor suppressor	Down	91/91	18S rRNA	[54]
SNORD50A SNORD50B	6q14.3	SNHG5	Tumor suppressor	Down	-	K-Ras	[62]
SNORD113-1	14q32.31	MEG8	Tumor suppressor	Down	112/112	ERK1/2, SMAD2/3	[63]
SNORA23	11p15.4	IP07	Tumor suppressor	Down	-	28S rRNA	[64]

The expression of snoRNA with sample size information was verified in HCC and normal liver tissues.
